# CAIX furthers tumour progression in the hypoxic tumour microenvironment of esophageal carcinoma and is a possible therapeutic target 

**DOI:** 10.1080/14756366.2018.1475369

**Published:** 2018-06-04

**Authors:** Astrid Drenckhan, Morton Freytag, Claudiu T. Supuran, Guido Sauter, Jakob R. Izbicki, Stephanie J. Gros

**Affiliations:** a Department of General, Visceral and Thoracic Surgery, University Medical Center Hamburg-Eppendorf, Hamburg, Germany;; b Department Neurofarba, Section of Pharmaceutical Sciences, University of Florence, Florence, Italy;; c Department of Pathology, University Medical Center Hamburg-Eppendorf, Hamburg, Germany;; d Department of Pediatric Surgery, Ûniversity Children’s Hospital Basel, Basel, Switzerland

**Keywords:** Esophageal carcinoma, hypoxia, carbonic anhydrase IX, tumour microenvironment, targeted therapy

## Abstract

The hypoxic tumour microenvironment of solid tumours represents an important starting point for modulating progression and metastatic spread. Carbonic anhydrase IX (CAIX) is a known HIF-1α-dependent key player in maintaining cell pH conditions under hypoxia. We show that CAIX is strongly expressed in esophageal carcinoma tissues. We hypothesize that a moderate CAIX expression facilitates metastases and thereby worsens prognosis. Selective inhibition of CAIX by specific CAIX inhibitors and a CAIX knockdown effectively inhibit proliferation and migration *in vitro*. In the orthotopic esophageal carcinoma model, the humanized HER2 antibody trastuzumab down-regulates CAIX, possibly through CAIX’s linkage with HER2 in the hypoxic microenvironment. Our results show CAIX to be an essential part of the tumour microenvironment and a possible master regulator of tumour progression. This makes CAIX a highly effective and feasible therapeutic target for selective cancer treatment.

## Introduction

Cancer cells are characterized by dysregulated cell proliferation. The blood vessels that form within solid tumours are structurally and functionally abnormal displaying often a reduced capillary density resulting in severe hypoxia^1^. Adaptation of cancer cells to the hypoxic microenvironment is regulated through physiological responses to hypoxia that are mediated by hypoxia-inducible factors, namely HIF-1α and HIF-2α. Due to this adaptation, hypoxic cancer cells acquire invasive and metastatic properties as well as resistance to chemotherapy and radiation therapy, which together constitutes the lethal cancer phenotype^1^. Carbonic anhydrase IX (CAIX) is a HIF-1α-dependent protein that regulates intra- and extra-cellular pH homeostasis under hypoxia. It is involved in numerous pathological processes including tumourgenicity^2^ and supposedly in malignant transformation^3^. The (over)-expression of CAIX has also been associated with poorer survival or prognosis in several other solid tumours such as oral squamous cell or gastric cancer^2^^,^^4–7^. Furthermore, CAIX has been linked with increased invasion, giving rise to the hypothesis that increased CAIX expression may, through invasion, contribute to advanced disease and tumour progression^3^.

We have previously investigated the roles of CAIX and PGK1, both enzymes that are controlled by HIF-1α and are up-regulated in the hypoxic microenvironment in neuroblastoma. We could recently show that CAIX expression in neuroblastoma cell lines is hypoxia-dependent and these cells are responsive to anti-CAIX treatment *in vivo*^8^. Our research indicates that PGK1 together with CXCR4 enhances metastases and that the presence of high CAIX expression is a negative prognostic factor in neuroblastoma patients^8^^,^^9^. A similar hypoxia-dependent expression of CAIX has been described for many solid tumours including esophageal cancer^6^^,^^7^^,^^10^. Expression of CAIX has been linked to a malignant phenotype^7^, adverse clinicopathological factors^11^, tumour cell dissemination^12–14^ and poor survival^2^^,^^4–7^. In breast cancer, CAIX was shown to be vital for growth and metastasis of hypoxic tumours and has been suggested as a specific and targetable biomarker for metastasis^13^^,^^15^.

Esophageal carcinoma affects more than 450,000 people worldwide, and the incidence, especially of that of adenocarcinoma in Western countries is rapidly increasing. The overall five-year survival of patients with esophageal carcinoma is still very poor and ranges from 15 to 25%^16^. Despite the efforts to improve combined surgical approach/radiochemotherapy, there is an immense clinical need for new therapeutic strategies and molecular targets^17^. Through modern technology, particular molecular profiles can be identified and novel targets can be developed for subgroups of patients^18^. For example, one of these developing targets has been the antibody trastuzumab, which has recently been approved for treatment of advanced gastric and gastroesophageal junction cancers^17^ and has shown efficacy in an orthotopic model of esophageal adenocarcinoma^19^^,^^20^.

The current study investigates the impact of hypoxia-dependent CAIX expression on proliferation, migration, and metastases *in vitro*, *in vivo,* and in our patient collective. The results will define the influence of hypoxia-dependent CAIX expression on tumour progression and strengthen its potential role as a clinical therapeutic target in a selected patient collective. Targeting of CAIX could become an additive treatment strategy for esophageal cancer, especially in advanced stages with limited treatment options.

## Methods and materials

### Cell lines and culturing conditions

Cell lines PT1590, LN1590, PT6216, LN6216c, LN6216gc, and LN6216o were acquired from primary tumour and lymph nodes of patients with esophageal adenocarcinoma at the University Medical Center Hamburg–Eppendorf as described previously^21–23^. OE19 and OE33 were purchased (Sigma-Aldrich, St. Louis, MO, USA). Aliquots of early passages (4–6) were used for all experiments. Cells were cultured in RPMI 1640 (Gibco, Thermo Fisher Scientific Inc., Waltham, MA, USA) with 10% fetal bovine serum (Gibco, Thermo Fisher Scientific Inc., Waltham, MA, USA), 10 μmol/ml transferrin (Sigma-Aldrich, St. Louis, MO, USA), 1 μg/ml insulin (Sigma-Aldrich, St. Louis, MO, USA), 1 μg/ml fibroblast growth factor (Peprotech, Hamburg, Germany) and 1 μg/ml epidermal growth factor (Peprotech, Hamburg, Germany). All cells were cultured in a humidified atmosphere at 37 °C either in air with 5% CO_2_ under normoxic or with 5% CO_2_/5% O_2_ balanced with N2 under hypoxic conditions.

### Reverse transcription quantitative PCR (RT qPCR)

Total RNA was isolated with RNeasy Mini Kit (Qiagen, Hilden, Germany) in accordance to the manufacturer´s protocol and reversely transcribed with Quantitect Reverse Transcription Kit (Qiagen, Hilden, Germany). CAIX and 18S specific primers (CAIX: Cat. No. PPH01751A; 18S: Cat. No. 330001 PPH05666E, Qiagen, Hilden, Germany) were used for amplification of cDNA, which was detected with Maxima SYBR Green (Thermo Fisher Scientific Inc., Waltham, MA, USA) in a Lightcycler 4800 (Roche, Penzberg, Germany). Patient tissue was analyzed in duplicates and triplicates, cell culture data in duplicates, *in vivo* data included tumour RNA of all animals of each treatment group (*n* = 9/10, respectively). Data were analyzed using the 2°(-Delta Delta C(T)) method as previously described^24^.

### Western blot analysis

Cells were grown to confluence under either normoxic or hypoxic conditions. Confluence was reached between 48 and 72 h. We did not observe a difference between normoxic and hypoxic conditions in reaching confluence. They were lysed with radioimmunoprecipitation assay buffer (Sigma-Aldrich, St. Louis, MO, USA). The protein detection was performed using the BCA Protein Assay Kit (Thermo Fisher Scientific Inc., Waltham, MA, USA). 20 µg of protein was dissolved on a 10% SDS-polyacrylamide gel electrophoresis and blotted to nitrocellulose membranes. After blocking with 5% dried milk for 1 h at room temperature, immunoblotting against CAIX was done with M75 antibody (BioScience Slovakia, Bratislava, Slovak republic) diluted 1/2000. Anti-α-Tubulin antibody (Cell Signaling Technology, Danvers, MA, USA) diluted 1/1000 served as a loading control. Super Signal West Dura Extended Duration Substrate (Thermo Fisher Scientific Inc., Waltham, MA, USA) was used for detection.

### Immunocytochemistry

Ten thousand cells were seeded in each chamber of a chamber slide (Thermo Fisher Scientific Inc., Waltham, MA, USA) and grown overnight to attach. They were fixed and permeabilized with 4% paraformaldehyde in phosphate-buffered solution. Blocking was done with 3% bovine serum albumin in phosphate-buffered solution with Tween 20 for 1 h at room temperature. M75 diluted 1:100 (BioScience Slovakia, Bratislava, Slovak republic) was used as the primary antibody against CAIX, detection was done with Alexa fluor 488 linked goat anti-mouse antibody (Invitrogen, Thermo Fisher Scientific Inc., Waltham, MA, USA) diluted 1/1000. VectaShield mounting medium with DAPI (Vector Laboratories, Burlingame, CA, USA) was used for DNA staining and mounting. The images were made with an Axio Scope A1 fluorescence microscope with AxioCam Mrc5 (Zeiss, Oberkochen, Germany).

### Tissue microarray (TMA) construction

Tissue samples were fixed in 4% buffered formalin, paraffin embedded, and used for TMA construction as previously described^25^. Briefly, hematoxylin-eosin stained sections were made from selected primary tumour blocks (donor blocks) to define representative tumour regions. Tissue cylinders (0.6 mm in diameter) were then punched from that region of the donor block using a home-made semi-automated tissue arrayer. 3 μm sections were made by use of the Paraffin Sectioning Aid System (Instrumentics, Hackensack, NJ, USA).

**Figure 1.**
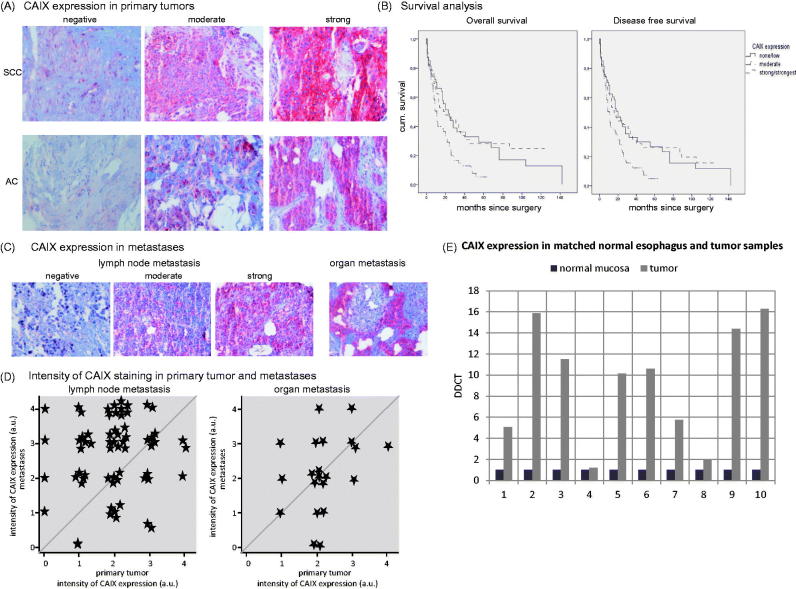
Esophageal carcinoma tissue and microarray. (A) Tissue micro array (TMA) of esophageal carcinoma primary tumour and metastatic tissue was examined for CAIX expression and grouped into negative, moderate, and strong CAIX expression. Figure 1(A) shows representative samples of immunohistochemical CAIX staining for adenocarcinoma and squamous cell carcinoma at a standard magnification of ×20. (B**)** Patients with a moderate CAIX expression had a significantly worse overall (*p* = .003 and *p* = .009) and disease free (*p* = .006 and *p* = .015) survival compared to patients with no or a strong CAIX expression. (C,D**)** CAIX expression of primary tumour was correlated with CAIX expression of lymph node and organ metastases (representative immunohistochemical stainings are shown in Figure 1(C). Figure 1(D) shows the correlation of the intensity of CAIX staining (0-none, 1-low, 2-moderate, 3-strong, 4-strongest) of primary tumour and metastases. The left graph depicts the intensity of CAIX staining of the lymph node metastases, the right graph shows the intensities of the organ metastases in correlation to the intensity score of their primary tumour. Interestingly, most lymph node metastases occurred in the group with a moderate CAIX expression and thus impaired prognosis (group 2 in Figure 1(D)) and the metastatic lymph nodes of these patients overall expressed CAIX in higher intensities than any of the others. (E) All tumour samples (*n* = 10), although to a different extent, showed an increased expression of RNA levels in quantitative PCR compared to their respective normal mucosal control tissues.

For the matched primary tumour and metastases analysis (Figure 1(D)), only patients whose metastatic tissue was present on the TMA were included. Although all primary tumours of the patient collective were present on the TMA, metastatic tissue of all metastatic patients was not. The metastatic samples for the TMA preparation were chosen in respect to their availability (amount of tissue).

### Immunohistochemistry

For the immunohistochemistry of the paraffin-embedded TMA, the HRP-ACE-System from R and D Systems (Minneapolis, MN, USA) were used. Sections were counterstained with Mayer’s hematoxylin solution (Merck, Burlington, NJ, USA). Tumour tissue was identified by hematoxylin eosin (HE) staining. The CAIX staining was performed using the primary antibody M75 (BioScience Slovakia, Bratislava, Slovak republic) at a dilution of 1:200. Control sections were incubated with antibody diluent (DAKO, Glostrup, Denmark) without primary antibody at 4 °C overnight and then treated as other samples. The scoring was performed according to a modification of the scaling system used for clinical scoring of molecular markers combining a score for area and intensity by two independent blinded examiners pathologically trained senior scientist (AD) and IHC experienced surgeon (SJG). For analysis, a BX43 Olympus Microscope was used and images were recorded using Cell Sense Software (Olympus, Volketswil, Switzerland) at a standard magnification of ×20.

### Patients

A patient collective of 161 patients, that underwent surgery in curative intention in the Department of Surgery at the University Medical Center Hamburg–Eppendorf, was examined for CAIX-expression by immunostaining. This study was approved by the ethics committee of the chamber of physicians at Hamburg, Germany and written consent was obtained from all patients to use the resected samples. Upon histopathological examination, the resection margins were tumour free. Patients had not been subjected to neoadjuvant treatment prior to operation. Tumour stage and grade were classified according to the tumour-node metastasis classification of the International Union Against Cancer^26^. Further, 10 matched patient samples of healthy esophageal mucosa and esophageal cancer from our cryo tissue bank were analyzed by PCR for CAIX expression. Samples were chosen on account of quality of tissue and availability.

### Cell proliferation assay

Cells were seeded at 5000 cells/well in 96-well plates. After 24 h of incubation, CAIX inhibitors FC5–207A and FC8–325A (Table 1)^8^ were added to the cells at final concentrations of 500 µM. Cell culture medium was used as control. Cells were then cultured under hypoxic or normoxic conditions. The MTT assay (CellTiter 96 Aqueous One Solution Cell Proliferation Assay, Promega, Madison, WI, USA) was carried out in accordance to the manufacturer’s protocol at 48 and 72 h. Absorbance was measured at 490 nm (FLUOStar Omega, BMG Labtech Inc., Cary, NC, USA). Each experiment was performed at least four times.

**Table 1.** Chemical Structures of CAIX Inhibitors.

FC5-207A	FC8-325A
	

The CA inhibitory data are found in reference [64].

**Table 2.** Patient characteristics.

Characteristic		Total (*n* = 161)	None/low CAIX expression	Moderate CAIX expression	Strong/strongest CAIX expression
Age	Years	61.86 (±9.371)	62.6 (±10.860)	61.56 (±8.643)	61.35 (±8.477)
Gender	Male	123	41	50	32
	Female	38	13	14	11
T-staging	1	26	12	6	8
	2	52	16	23	13
	3	80	26	33	21
	4	3	0	2	1
N-staging	0	66	24	21	21
	1	95	30	43	22
M-staging	0	137	48	49	40
	1	24	7	14	3
Grading	1	2	0	1	1
	2	93	32	29	32
	3	66	22	34	10
Cell type	SCC	89	28	35	26
	AC	69	26	26	17
	ASQ	3	0	3	0

SCC: squamous cell carcinoma; AC: adeno carcinoma; ASQ: adeno squamous carcinoma.

### Knock-down of CAIX

PT6216 cells were transduced with a lentiviral vector under appropriate local security protocols. The plasmid contains siRNA against CAIX mRNA and GFP coupled with puromycin resistance under the same promotor region (Cat. No. I000160, Applied Biological Materials Inc, Richmond, Canada). HEK293T cells were transiently transfected to produce viral supernatants as described^27^. The following packaging and envelope plasmids were used: pMDLg/pRRE, pRSV-Rev and VSV-G (kindly provided by B. Fehse, Research Laboratory for Cell and Gene Therapy, Department for Stem Cell Transplantation, University Medical Center, Hamburg-Eppendorf, Germany). Target cells were transduced following the protocol as described^28^. Cells showing the highest down-regulation of CAIX in Western blot analysis were chosen for cell viability and cell proliferation assays. Transfected cells were selected with puromycin and cultured for two weeks before use.

### Cell migration assay

Tumour cell migration through a microporous membrane was assessed using a Boyden transwell system (8 µl pore size, Corning Costar, NY, USA). Before seeding transwell, systems were incubated in RPMI-media containing 2% FCS for 30 min at room temperature. 200 µl RPMI-media with 2% FCS containing dissociated cells (3 × 10^4^ per well) was added to the upper insert of the chamber. In the bottom chamber, 500 µl RPMI-media with 10% FCS was added. Twenty four hours after seeding, the transwell systems were transferred to a new plate containing 500 µl trypsin (Sigma-Aldrich, Munich, Germany) and incubated at 37 °C for 30 min to remove cells attached to the bottom side of the membrane. After incubation, the transwell systems were removed and the trypsin solution containing migrated cells were centrifuged. Cells were stained with calcein (Invitrogen, Carlsbad, CA, USA) for 30 min at 37 °C. After two washing steps, calcein absorbance was measured at 485 nm (FLUOStar, Omega, BMG Labtech, Ortenberg, Germany). Each experiment was repeated at least six times.

### Orthotopic implantation and* in vivo* treatment

Orthotopic implantation in the esophageal implantation model was obtained as previously described using NMRI/nu mice (Charles River, Germany) and OE19 cell line. All animal procedures were performed in accordance with a protocol approved by the Behörde für Wissenschaft und Gesundheit (Freie und Hansestadt Hamburg, Germany). After primary tumour growth was established by magnetic-resonance-imaging (MRI) on day 14, mice were randomized into four groups of nine mice each (10 mice in the control group). Group one was treated biweekly with an intra-peritoneal injection of 20 mg/kg body weight trastuzumab (Roche, Penzberg, Germany) in a volume of 100 μl. Group two received 5 mg/kg body weight AMD3100 (Sigma-Aldrich, Munich, Germany) in 100 ml by intra-peritoneal injection. Group three received 25 mg/kg body weight CTCE-9908 (British Canadian BioSciences Corp) in 100 ml by intra-peritoneal injection daily. The control group was for given daily intra-peritoneal sham injections with 100 ml PBS.

### Statistical analysis

The statistical analysis was conducted using SPSS version 13.0 (SPSS, Chicago, IL, USA). *p* Values less than .05 was defined as significant. Kaplan–Meier survival analysis and log-rank test were performed to compare the survival time between groups. For significance testing, the two-sided *t*-test was used for *in vitro* experiments. Normal distribution of the measured values was proven before by quantile-quantile plots and Shapiro–Wilk test of normality (data not shown). The error bars in all bar plots represent one standard deviation.

## Results

### Hypoxic microenvironment of esophageal carcinoma

We hypothesized that in the hypoxic tumour environment, CAIX expression influences tumour progression and metastases of esophageal cancer to a great extent. In order to investigate, if CAIX is highly expressed in a relevant number of carcinoma patients and this expression is of consequence for the clinical outcome, we evaluated a TMA of esophageal carcinoma tissues by immunohistochemical staining (Figure 1(A), Table 2).

**Figure 2.**
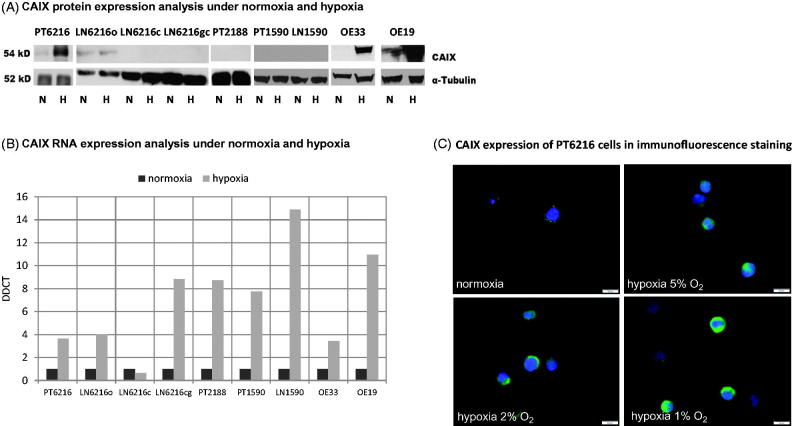
CAIX expression in esophageal carcinoma cell lines. Protein (A) and RNA (B) levels of CAIX expression of esophageal carcinoma cell lines are shown under normoxic and hypoxic conditions. DDTC was calculated using 2^(-Delta Delta C(T)) method^24^. (C**)** Hypoxia-dependent expression of CAIX in PT6216 cells under normoxic and hypoxic conditions with 5%, 2%, and 1% O_2_ respectively.

It has previously been shown that expression of CAIX is associated with a poorer overall and disease-free survival in esophageal carcinoma^6^^,^^7^^,^^10^. In our patient collective, we were able to identify a subgroup of patients with a moderate CAIX expression that had a severely impaired prognosis (Figure 1(B)). This was also the group with the highest metastatic rate regarding both lymph node and organ metastases (Figure 1(C)) at the time of operation. Lymph node metastases of these patients overall expressed CAIX in higher intensities than any of the others (Figure 1(C,D)). CAIX expression in this patient collective was independent of clinical stage, grading, or age (data not shown).

Normal esophagus and esophageal carcinoma tissue from the same organ of more recently operated patients were examined for CAIX RNA expression by quantitative PCR (Figure 1(E)). CAIX was up-regulated in all carcinoma tissues compared to their matching normal control. The number of samples is too low to draw conclusions from the data regarding a possible relevance of the degree of CAIX up-regulation. Although the extent of up-regulation varied between the patients, CAIX appears to play a crucial role in all tumour tissues regardless of cell type.

### Hypoxia-dependent CAIX expression of esophageal tumour cells

**Figure 3.**
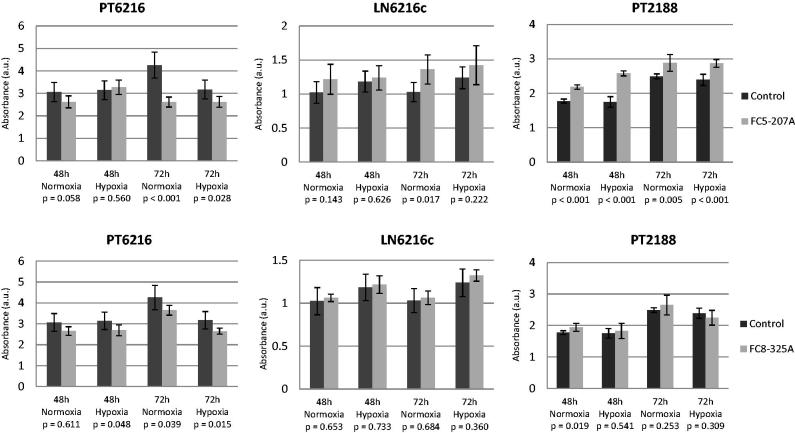
Inhibition of proliferation of esophageal carcinoma cell line PT6216, LN6216c, and PT2188. The specific CAIX inhibitors FC5–207A and FC-8–325A were used to inhibit CAIX *in vitro. S*ignificant decreases in proliferation were observed under normoxic and hypoxic conditions for both inhibitors for cell line PT6216 (left panels). Cell lines LN6216c and PT2188 do not express CAIX and neither inhibitor led to a decrease in proliferation, but rather, although this effect was not consistently significant to an increase of proliferation (middle and right panels). Absorbance (AU) is represented on the y-axis.

Several esophageal carcinoma cell lines were examined regarding the expression of CAIX protein and RNA. Cells were exposed to normoxic or hypoxic conditions. Although CAIX RNA was up-regulated in almost all cell lines under hypoxic conditions (Figure 2(B)), CAIX protein was only significantly increased in PT6216, OE33, OE19 cell lines (Figure 2(A)). The results of the Western blot were confirmed by immunocytochemistry as exemplarily shown for cell line PT6216 (Figure 2(C)). It is interesting here that, although cell line PT6216, which was generated from the primary tumour, showed significant expression of CAIX under hypoxia, its matching cell lines, that were derived from lymph nodes of the same patient, did not. Although CAIX mRNA was up-regulated under some conditions after 72 h, e.g. PT2188 (Figure 3), there is a lack of anti-proliferative response to CAIX inhibition in cell lines that did not up-regulate CAIX protein after 48 h.

### CAIX-dependent proliferation and targeted therapy

To investigate the influence of CAIX on tumour cell proliferation, we again exposed PT6216, LN6216c, and PT2188 to hypoxic and normoxic conditions. We used the two specific CAIX inhibitors, FC5–207A and FC-8–325A to inhibit CAIX *in vitro* in these cell lines in a MTT assay. After treating PT6216 cells, which express low levels of CAIX under normoxia and elevated levels under hypoxia, for 72 h with CAIX inhibitor, significant decreases in proliferation were observed under normoxic and hypoxic conditions for both inhibitors (Figure 3, left panels). In cell lines LN6216c and PT2188, which do not express CAIX under normoxia or hypoxia, neither inhibitor led to a decrease in proliferation. Although this effect was not consistently significant, treatment rather led to an increase of proliferation (Figure 3, middle and right panels).

**Figure 4.**
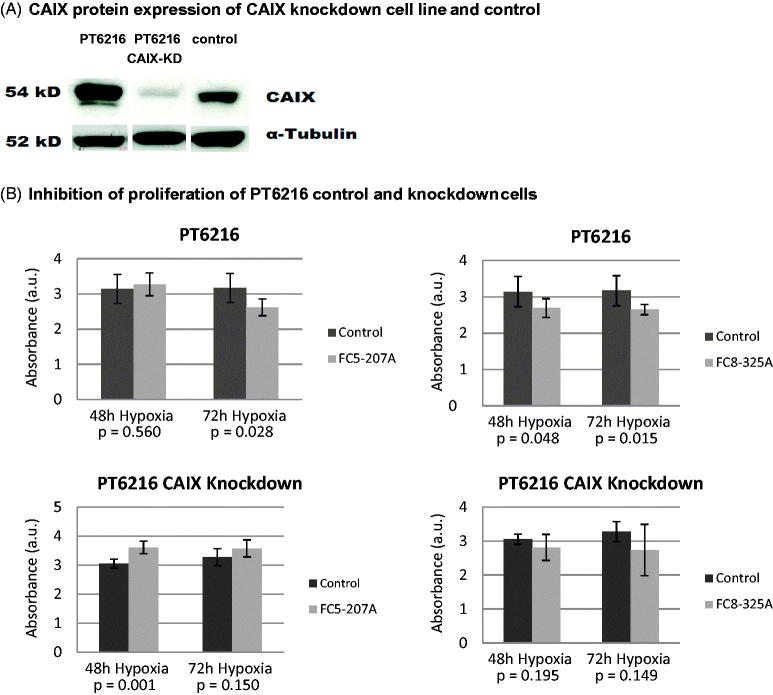
CAIX Knockdown in esophageal carcinoma cell line PT6216. (A) RNA results were verified by analysis of CAIX protein expression. (B) Inhibition of the PT6216 control cells led to a significant decrease of proliferation after 72 h treatment with both inhibitors under hypoxia (upper panels), but was not effective in the equivalent CAIX-knockdown cells (lower panels). Absorbance (AU) is represented on the y-axis.

To verify these results, we constructed a CAIX knockdown of cell line PT6216 that showed minimal residual CAIX protein expression (Figure 4(A)). Exposure to hypoxia did not lead to an increase in proliferation of PT6216 CAIX knockdown cells (data not shown). While inhibition of the PT6216 control cells led to a significant decrease of proliferation after 72 h treatment with both inhibitors under hypoxia (Figure 4(B) upper panels), anti-CAIX treatment was not effective in the equivalent CAIX-knockdown cells (Figure 4(B) lower panels).

### CAIX-dependent migration* in vitro *and impact of CAIX expression in the metastatic process *in vivo*

**Figure 5.**
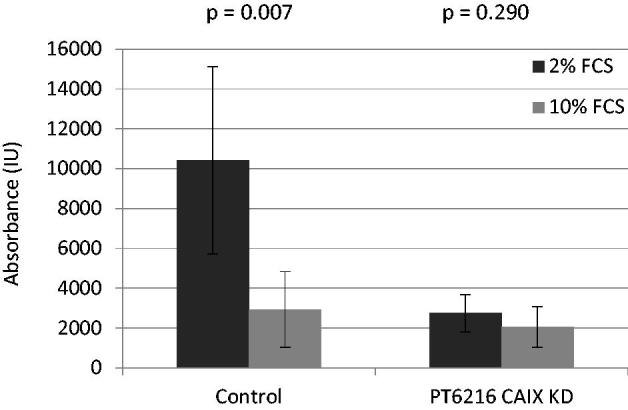
Migration of PT6216 control and knockdown cells *in vitro***.** Migration *in vitro* assays showed that while regular PT6216 cells migrate under exposure to 2% FCS as chemotactic stimulant the CAIX knockdown cells lost the ability to migrate.

In order to investigate the effect of CAIX expression on migration, *in vitro* migration assays were carried out with PT6216 control and PT6216 CAIX knockdown cells using a FCS gradient as migration stimulus. The results show that while regular PT6216 cells migrate under exposure to 2% FCS as chemotactic stimulant, the CAIX knockdown cells lost the ability to migrate (Figure 5).

**Figure 6.**
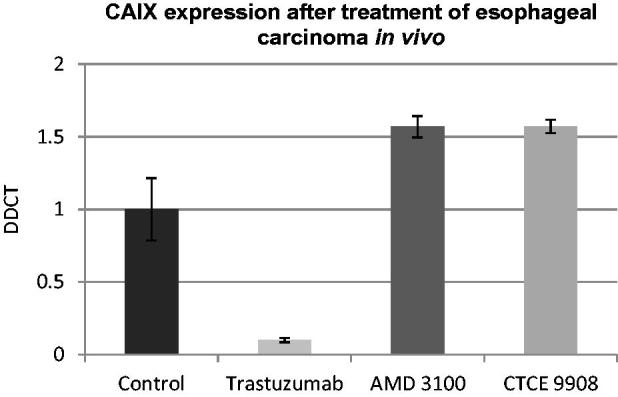
CAIX expression after treatment of esophageal carcinoma *in vivo.* While treatment showed a significant decrease in tumour progression in all treatment groups, as previously published^19^^,^^20^^,^^29^, only trastuzumab led to a suppression of CAIX, while treatment with both CXCR4 inhibitors led to an up-regulation of CAIX. Standard deviation was calculated as percent deviation of Delta-CT values.

Further insight into CAIX expression under anti-tumour treatment *in vivo* can be gained from the orthotopic esophageal carcinoma implantation model. CAIX RNA of control primary tumours was obtained to define standard CAIX levels in these tumours. Three further groups were treated with trastuzumab, an antibody against HER2, as well as AMD3100 and CTCE9908, two inhibitors of CXCR4 that have been shown to be involved in migration and tumour cell homing in esophageal carcinoma. Treatment with trastuzumab led to a suppression of CAIX, while treatment with both CXCR4 inhibitors led to an up-regulation of CAIX (Figure 6). In all treatment arms, as previously shown, a significant decrease of tumour progression could be observed^19^^,^^20^^,^^29^.

## Discussion

### CAIX in the hypoxic microenvironment

The microenvironment as well as the intracellular compartment of many solid tumours is often hypoxic as neovascularization does not keep up with tumour growth. While several cellular factors are important for metastases, the tumour’s microenvironment also plays a crucial role in the metastatic process^30–32^. To make survival in this hypoxic and acidic environment possible, tumour cells react with a modified gene expression^33–37^. For example, under hypoxia, one of the key response regulators HIF-1α can build dimers with HIF-1β, migrate into the cell nucleus, and bind to promoters of its target genes. Most target genes of HIF-1α, like CAIX, have a hypoxia-response-element (HRE)^38^. The transmembrane metallo-enzyme CAIX is a target gene of HIF-1α and plays a key role in cell survival in the acidic tumour microenvironment^30^^,^^39^^,^^40^. CAIX maintains the intracellular physiological pH 7.2 and at the same time, lowers the extracellular pH through extracellular CO_2_-derived protons^13^^,^^41^. The acidic extracellular pH facilitates migration of malignant cells from the primary tumour and formation of metastases^40^^,^^42^. Moreover, tumour hypoxia is often associated with highly aggressive tumour growth and radio- and chemoresistance^43^.

### Influence of the hypoxic environment *in vitro *and *in vivo*

We could show here that the hypoxic microenvironment plays a major role in tumour proliferation and metastases. Our patient data suggest that a moderate CAIX expression furthers tumour proliferation and metastases resulting in a worse prognosis in these patients. It also can be observed that CAIX expression mostly increases in metastatic tissue. One hypothetical explanation for this phenomenon could be that a specific subpopulation with higher CAIX expression is responsible for metastatic spreading and homing. Another hypothesis could be that once tumour cells are homed to their target organ, they change their CAIX expression profile to a higher CAIX expression in adaptation to the new microenvironment. Analysis of the paired esophagus samples (tumour and normal tissue) impressively suggests an enormous importance of this hypoxia-regulated factor in tumour development and progression as well as an enormous potential as a treatment target.

Several esophageal cell lines express CAIX. We show here that CAIX expression is induced by hypoxia confirming the regulatory importance of the microenvironmental conditions. Through inhibition of CAIX with CAIX inhibitors FC5–207A and FC8–325A and a selective CAIX knockdown cell line, we could show that anti-CAIX treatment is effective in CAIX-positive esophageal carcinoma *in vitro*.

While cell line PT6216 and LN6216c are established from the same patient with advanced disease, only the primary cell line expresses CAIX while the lymph node metastasis is negative. This is consistent with the results of the proliferation assay while PT6216 is responsive to inhibition of CAIX, LN6216c is not. Cell lines LN6216c and PT2188, that both do not up-regulate CAIX protein, do not respond with a decrease of proliferation when treated with the inhibitor FC8–325A. When treated with the inhibitor FC5–207A cell line, PT6216c shows an increased proliferation under hypoxic but not normoxic conditions after 72 h. In cell line PT2188, this effect is observed under all conditions. It could be hypothesized that in the absence of CAIX, the inhibitor FC5–207A induces a proliferative process in PT2188 cells that is yet unobserved. In both cell lines LN6216 and PT2188, no increase of CAIX protein is observed. However, cell line PT6216, which up-regulates both CAIX mRNA and protein is responsive to both inhibitors under hypoxic conditions. To further validate that this effect is regulated by CAIX, we constructed the CAIX knockdown cell line and are able to confirm our results.

The finding that primary tumour and metastases might react differently to therapy sheds light on the difficulties that clinicians face in treating esophageal carcinoma if surgery is not an option and resistance to treatment becomes obvious. There is great necessity to further investigate and understand tumour heterogeneity.

We further investigated the impact of CAIX on tumour cell migration and found that a knockdown of CAIX results in the loss of the cells’ migratory potency. This suggests a crucial role of CAIX for metastatic spread in esophageal carcinoma as well as a potential therapeutic approach.

### Interaction of CAIX with other relevant factors that enhance proliferation and migration

In the orthotopic esophageal model inhibition of the HER2 receptor with the humanized antibody trastuzumab impressively leads to a reduction of CAIX expression as well as significant reduction of proliferation and metastases as previously shown *in vivo*^19^^,^^20^. It can be hypothesized that CAIX as part of the hypoxic microenvironment and HER2 share phenotypic and genetic components that are modulated through HIF-1α regulation, which results in synergistic effects upon inhibition of one component, in this case HER2. It has been shown through gene expression profiling of hypoxic mammary tumours that known targets of the HER2/neu pathway are elevated, which suggests that hypoxic condition and HER2 overexpression share phenotypic and genetic components through HIF-1α regulation^44^. It was further demonstrated that HER2 requires HIF-1α to induce proliferation and is suggested to be a major downstream regulator of this pathway. In smooth muscle cells, it could be shown that intermittent but not sustained hypoxia increases HER2 expression and cell proliferation^45^. A recently published study on breast cancer showed that progesterone-induced signaling triggers migration of cancer cells from early lesions shortly after HER2 activation, but promotes proliferation in advanced primary tumour cells^46^. They found that the switch from migration to proliferation was regulated by increased HER2 expression and tumour-cell density involving microRNA-mediated progesterone receptor down-regulation^46^. A correlation of HER2 with CAIX was previously described for esophageal carcinoma. The authors hypothesize that the induction of a “hypoxic phenotype”, independent of hypoxia, through amplifying the cellular response could be a possible explanation for the association of HER2 and CXCR4^6^. Our data further support this as HER2-targeted therapy in these HER2 overexpressing tumours inhibits tumour progression and metastases and furthermore, leads to a decrease of CAIX expression, which may further augment this process.

HIF-1α also plays a crucial role in regulation of CXCR4/SDF-1α axis in migration to ischemic tissues^47^. Activated through hypoxia, HIF-1α acts as an important regulator of CXCR4^48^^,^^49^. In patients with chronic lymphatic leukemia HIF-1α correlates with CXCR4 expression and appears to have a relevant role in pathogenesis^50^. A correlation of these two factors has been confirmed for various cancers including breast cancer, osteosarcoma, glioma, glioblastoma, and neuroendocrine tumours^51–56^. Migrated endothelial progenitor cells can, for example, secrete SDF-1α themselves to recruit the additional migration of CXCR4 positive PECs or inflammatory cells to the site of ischemic tissues, suggesting that they function through a paracrine mechanism^47^. While it has been established that HIF-1α is a key regulator of CXCR4 expression, our results show that selective inhibition of CXCR4 does not lead to a down-regulation of HIF-1α’s hypoxia-inducible downstream target CAIX. Rather interestingly, inhibition of CXCR4 increases expression of CAIX, suggesting that inhibition of CXCR4 does not necessarily lead to reduction of HIF-1α. A further explanation could be that the CXCR4 - HIF-1α (-CAIX) axis is more prone to interference by other factors or more receptive to counter regulatory mechanisms. This theory can be supported by the observation that the CXCR4 ligand SDF-1α, which was shown to be induced amongst others by acid in principal cells^57^, has been shown to protect hepatocytes from ischemic shock^58^.

Combining novel therapeutic approaches with gold standard therapies, one has to be aware of so far unknown interaction. For example, it has previously been shown that the combination therapy of a CAIX inhibitor, here the monoclonal antibody girentuximab, with the tyrosine kinase inhibitor led to severe issues regarding uptake and accumulation^59^. These issues, however, could be resolved after further studies on biodistribution^60^.

### Clinical relevance of CAIX

In esophageal carcinoma, it has previously been shown that overexpression of CAIX is associated with an impaired prognosis to different extents in adenocarcinoma and squamous cell carcinoma of the esophagus^10^^,^^61^. In a Japanese study, high expression of CAIX was associated with a poor prognosis and a malignant phenotype in patients with SSC of the esophagus^7^. In two further studies, a high CAIX expression was associated with shorter survival in both adeno and squamous cell esophageal carcinoma^6^^,^^61^. However, a positive correlation of CAIX with Her2 overexpression can only be observed in adenocarcinoma^6^. A study by Driessen et al. examined adenocarcinomas of the esophagus, cardia, and distal stomach and found CAIX to be an independent prognostic factor for overall survival^10^. High CAIX expression might be related to acidic microenvironment caused by GERD in gastroesophageal junction, associated with tumourigenesis^62^. Survival in this patient collective did not vary in adenocarcinomas in dependence of high or low CAIX expression. Overall, CAIX was more often found in glandular mucosa with or without dysplasia than in squamous epithelium or SCC^62^.

In our patient collective, presence of moderate or strong expression did not vary significantly between cell types. We could, however, identify a subset of patients with a moderate CAIX expression that significantly differed in their prognosis from CAIX-negative and cells expressing CAIX strongly. We correlated the CAIX expression of the primary tumours with the CAIX expression of the lymph nodes present on our tumour array. We found that the group with a moderate CAIX expression in the primary tumour presented with the most lymph node metastases. Although this, in part, represents a selection bias of the tissue micro array, it suggests that this group with a moderate CAIX expression might modulate a cell behaviour that leads to a more invasive and migratory phenotype. In the study by Birner et al., a correlation of the hypoxic phenotype in primary tumour and metastases, meaning its CAIX expression profile, was preserved at least during formation of lymph node metastases^6^. The question, however, remains why two metastases of the same patient would demonstrate different behaviour under hypoxia (Figure 2(A)). One possible explanation for this could be the artificial *ex vivo* environment. But, we also observed a variance in CAIX expression variance between primary tumour and lymph nodes in the patient samples, in which the metastasis has a lower CAIX intensity than the primary tumour (Figure 1(D)). Future research will be directed towards clarifying this question as well as to refining specificity of treatment options and their clinical applicability. These factors suggest an essential role of CAIX as part of the hypoxic microenvironment, which might result in the formation of aggressive subsets of tumour cells under mild hypoxia that lead to progression and metastatic spread in these cancers. Transferring our knowledge into the orthotopic metastatic esophageal carcinoma model will help addressing these questions.

In our study, we integrated several CAIX inhibitors that were designed with a focus on CAIX specificity and have been used in preclinical settings^63^^,^^64^. Further option of CAIX inhibition for future clinical application could be the monoclonal anti-CAIX antibody G250 (girentuximab) that has successfully been introduced in preclinical studies reducing tumour growth as well as enhancing several imaging modalities^65–68^. A phase one clinical trial using G250 could establish the importance of CAIX in renal cell carcinoma. A phase two clinical trial investigated the efficacy of lutetium 177-girentuximab radioimmunotherapy in patients with metastatic kidney cancer and resulted in disease stabilization^69^.

## Conclusion

We show that CAIX is strongly expressed in esophageal carcinoma tissues. A moderate CAIX expression appears to facilitate metastases and thereby worsens prognosis. Selective inhibition of CAIX by specific CAIX inhibitors and a CAIX knockdown effectively inhibit proliferation and migration *in vitro*. In the orthotopic esophageal carcinoma model, the humanized HER2 antibody trastuzumab down-regulates CAIX, possibly through CAIX’s linkage with HER2 in the hypoxic microenvironment. Our results show that CAIX, as part of the hypoxic tumour microenvironment of esophageal carcinoma, represents an important starting point for modulating tumour progression and metastatic spread. This makes CAIX a highly effective and feasible therapeutic target for selective cancer treatment.
